# Study on the sleep–wake circadian rhythm and phenotypic characteristics in the acute phase of hemorrhagic stroke

**DOI:** 10.3389/fnins.2025.1633011

**Published:** 2025-11-06

**Authors:** Xiaodong Yuan, Yongshan Fu, Ya Ou, Jing Xue, Na Yang, Hongrui Liu, Tiantian Wang, Jing Wang, Cuiping Yan, Pingshu Zhang

**Affiliations:** 1 Department of Neurology, Kailuan General Hospital, North China University of Technology, Tangshan, Hebei, China; 2 Key Laboratory of Neurobiological Function in Hebei Province, Tangshan, Hebei, China

**Keywords:** stroke, cerebral hemorrhage, sleep–wake, circadian rhythm, sleep disorder

## Abstract

**Objective:**

To investigate the sleep–wake circadian rhythm and phenotypic characteristics in patients with acute intracerebral hemorrhage (ICH), and to explore the relationship and potential mechanisms between sleep–wake phenotypes and circadian rhythm disruption.

**Methods:**

A retrospective analysis was conducted on 100 patients with acute ICH admitted to Kailuan General Hospital between January 2020 and December 2024, along with 67 age- and sex-matched hospitalized controls. Sleep parameters during the daytime (06:00–18:00) and nighttime (18:00–06:00) were collected using a mattress-based sleep monitoring system. Circadian rhythm indices—including interdaily stability (IS), intradaily variability (IV), and relative amplitude (RA)—as well as sleep phenotypic features were evaluated.

**Results:**

Compared with the control group, patients with ICH exhibited significantly disrupted circadian rhythms, characterized by reductions in IS, IV, and RA (all *p* < 0.05). Significant alterations in sleep–wake states were observed in both daytime and nighttime periods among ICH patients. These included prolonged/increased durations and proportions of light sleep, deep sleep, NREM sleep, REM sleep, and sleep efficiency, along with shortened/decreased sleep latency, REM latency, and proportion of NREM sleep (all *p* < 0.05). Phenotypic analysis revealed a significantly higher prevalence of excessive daytime sleep, increased total sleep across 24 h, and reversed sleep–wake cycles in the ICH group (all *p* < 0.05). Regression analysis indicated that reduced IS was significantly associated with reversed sleep–wake cycles (OR = −5.831; 95% CI,: −12.577 ~ −1.350).

**Conclusion:**

Acute hemorrhagic stroke is associated with excessive sleepiness, disrupted sleep architecture, and weakened circadian rhythms. These disturbances may impair recovery and long-term outcomes, underscoring the need for rhythm-based biomarkers and individualized interventions.

## Introduction

Intracerebral hemorrhage (ICH) accounts for over 10% of the approximately 17 million stroke cases worldwide each year. Although its incidence is lower than that of ischemic stroke, ICH is associated with a mortality rate exceeding 40%, and only about 20% of survivors regain functional independence within 6 months. This makes ICH one of the most lethal and difficult-to-treat subtypes of stroke (Broderick et al., 2021; Ohashi et al., 2023).

The circadian rhythm system plays a pivotal role in maintaining physiological homeostasis by synchronizing tissue-level functions with behavioral and environmental cycles such as sleep, activity, feeding, and light–dark rhythms. Disruption of circadian rhythms can lead to both acute and cumulative adverse health effects (Patke et al., 2020). The suprachiasmatic nucleus (SCN), located in the hypothalamus, acts as the central pacemaker of the circadian system. It transmits temporal cues to peripheral tissues via autonomic pathways, rhythmic secretion of melatonin from the pineal gland, and other efferent signals, thereby ensuring phase coherence across the body’s biological clocks (Zhou et al., 2022).

Among various circadian-regulated processes, the sleep–wake cycle is the most prominent in humans. This daily rhythm is governed by two opposing regulatory forces: the homeostatic sleep drive and the circadian wake drive (Dopp et al., 2024). The homeostatic drive increases with prolonged wakefulness and exposure to light, counteracting the circadian promotion of alertness. A well-regulated sleep–wake cycle is essential for brain function and overall health, offering neuroprotective benefits through several mechanisms, including reductions in metabolic rate and core temperature, modulation of inflammation and apoptosis, and enhancement of neuroplasticity—all of which contribute to neurological recovery (Pace et al., 2018).

These findings suggest that sleep may influence both the onset and clinical outcomes of stroke. However, existing research has predominantly focused on circadian rhythm disturbances and sleep architecture alterations in patients with ischemic stroke, exploring their potential prognostic implications (Miano et al., 2022; Wang et al., 2024).

Studies have shown that the 72-h period following the onset of intracerebral hemorrhage represents a high-risk time window for hematoma expansion and neurological deterioration, as well as a critical intervention period that determines patient prognosis (Lord et al., 2015; Zang et al., 2019; Anderson et al., 2010). Therefore, investigating alterations in the sleep–wake circadian rhythm during this crucial window is particularly important for understanding the mechanisms underlying central–peripheral rhythm coupling imbalance.

Studies investigating circadian and sleep–wake characteristics in patients with ICH, particularly during the acute phase, remain limited. Specifically, little is known about how the diurnal sleep–wake rhythm is affected in this population. Moreover, it remains unclear to what extent peripheral circadian rhythms can be autonomously maintained during the acute stage of cerebral hemorrhage.

The primary aim of this study is to comprehensively examine alterations in circadian sleep–wake patterns and sleep architecture in patients with ICH during the acute phase, and to characterize the specific manifestations of circadian rhythm disruption in this context.

## Materials and methods

### Participants

This was a retrospective study that included patients in the acute phase of intracerebral hemorrhage (ICH) who were diagnosed and treated in the Department of Neurology at Kailuan General Hospital and its affiliated Linxi Branch, both under North China University of Science and Technology, between January 2020 and December 2024.

Inclusion criteria: (1) Age between 18 and 75 years; diagnosis of ICH based on the 2019 Chinese Guidelines for the Diagnosis and Treatment of Cerebral Hemorrhage and confirmed by cranial computed tomography (CT) showing a clear hemorrhagic lesion; (2) No disturbances of consciousness. (3) A minimum of 3 days of continuous circadian sleep monitoring, with at least 16 h of data recording per day.

Exclusion criteria: (1) Traumatic brain hemorrhage, post-thrombolysis hemorrhage, or subarachnoid hemorrhage; (2) History of psychiatric disorders or cognitive impairment; (3) Severe dysfunction of major organs such as the heart, lungs, or liver; (4) Use of sedative or hypnotic medications.

A total of 148 patients were initially screened, including 104 males (70.2%) and 44 females (29.7%). Based on the inclusion and exclusion criteria, 100 patients were ultimately enrolled (72 males and 28 females), while 48 patients were excluded.

In addition, 67 hospitalized patients during the same study period were selected as the control group.

#### Inclusion criteria

(1) patients hospitalized in the same ward during the same period, aged 18–75 years; (2) absence of any acute cerebrovascular events at enrollment (including acute ischemic stroke, intracerebral hemorrhage, and subarachnoid hemorrhage); (3) continuous rest–activity rhythm monitoring for ≥3 days with at least 16 h of valid daily recording, using the same device and algorithms as in the case group; (4) exposure to identical nursing care and ward environment as the case group.

#### Exclusion criteria

**Figure 1:**
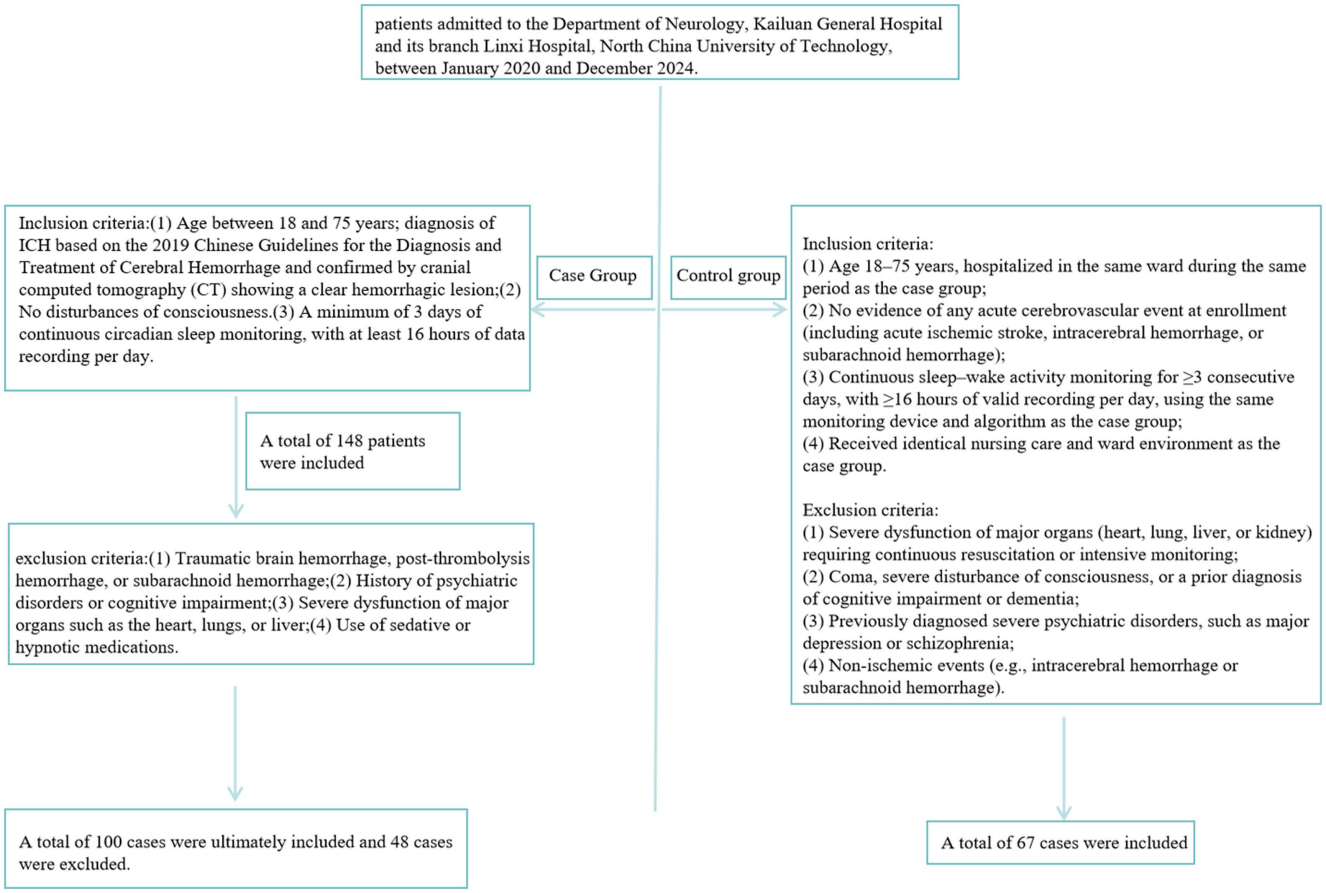
Grouping flowchart.

(1) severe dysfunction of vital organs (heart, lung, liver, kidney) requiring continuous rescue or intensive monitoring; (2) presence of coma, severe consciousness disturbance, or a prior diagnosis of cognitive impairment or dementia; (3) history of major psychiatric disorders, such as depression or schizophrenia; (4) non-ischemic cerebrovascular events (intracerebral or subarachnoid hemorrhage) (Figure 1).

### Baseline data

Demographic and clinical characteristics were collected, including sex, age, body mass index (BMI), medical history (hypertension, diabetes mellitus, hyperhomocysteinemia, hyperlipidemia), smoking status, and alcohol consumption history.

### Data collection

After admission, patients underwent continuous sleep–wake monitoring using the SC-500 mattress-based sleep monitoring system, covering both daytime (6:00–18:00) and nighttime (18:00–6:00) periods. This device is capable of detecting frequency-domain signals ranging from 0.01 to 10 kHz and can accurately separate vital signs such as heart rate (0.8–1.5 Hz) and respiration (0.2–0.8 Hz) from mixed signals (Liu et al., 2023). Furthermore, its monitoring accuracy has been validated in previous studies, demonstrating good agreement with polysomnography, the gold standard (Ding et al., 2022). Raw monitoring data were recorded daily on a cloud platform by trained personnel.

Based on these recordings, the following sleep parameters were extracted for both daytime and nighttime: total recording time, total sleep time, sleep latency, rapid eye movement (REM) sleep latency, wake after sleep onset, light sleep duration, deep sleep duration, non-REM (NREM) sleep duration, and REM sleep duration. Derived sleep metrics included:

- Proportion of light sleep = light sleep duration / total sleep time
- Proportion of deep sleep = deep sleep duration / total sleep time
- Proportion of REM sleep = REM sleep duration / total sleep time
- Proportion of NREM sleep = NREM sleep duration / total sleep time
- Sleep efficiency = total sleep time / total recording time

**Figure 2:**
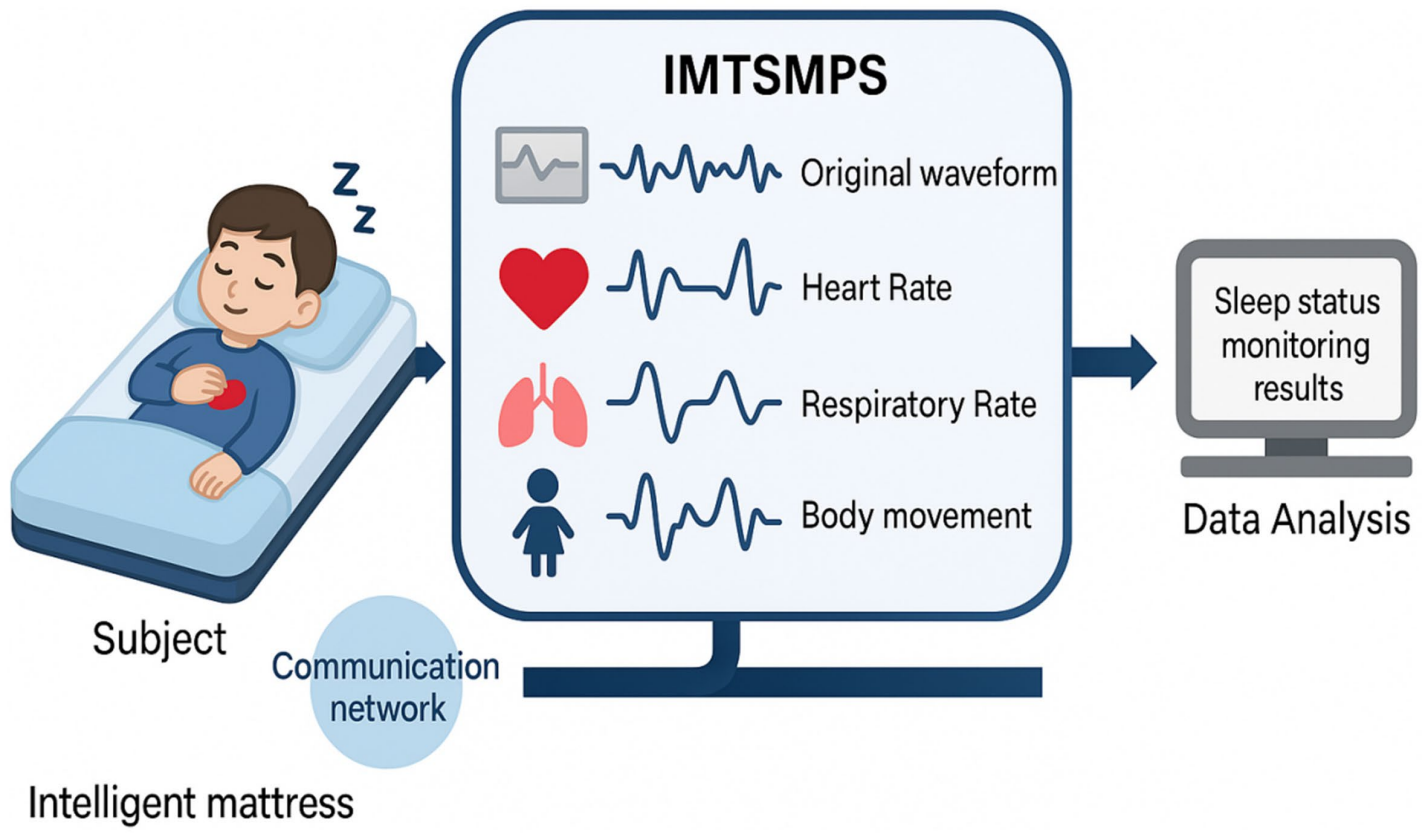
Monitoring process.

All sleep parameters were expressed as the average values across the total recording duration (Figure 2).

### Assessment of sleep–wake circadian rhythms

Circadian rhythm disturbances were assessed using three core metrics: inter-daily stability (IS), intra-daily variability (IV), and relative amplitude (RA).

- IS evaluates the consistency of 24-h activity-rest patterns and the synchronization between rest–activity rhythms and the external day–night cycle. IS values range from 0 to 1, with values closer to 1 indicating more stable circadian rhythms.
- IV reflects the fragmentation of the rest–activity rhythm, indicating the frequency and intensity of transitions between rest and activity within a 24-h period. Values range from 0 to 1, with higher values indicating more fragmented rhythms.
- RA is calculated based on activity levels during the most active 10-h period (M10) and the least active 5-h period (L5).

All parameters were derived from heart rate data obtained during sleep monitoring.


$$\text{IS}=\frac{p\sum_{h=1}^{p}{\left(x_{h}-\bar{x}\right)}^{2}}{p\sum_{i=1}^{n}(x_{i}-\bar{x})}\mathrm{IV}=\frac{n\sum_{i=2}^{n}{\left(x_{i}-{\bar{x}}_{i-1}\right)}^{2}}{(n-1)p\sum_{i=1}^{n}{\left(x_{i}-\bar{x}\right)}^{2}}$$

$$\mathrm{RA}=\frac{\left(M_{10}-L_{5}\right)}{\left(M_{10}+L_{5}\right)}$$

n: Total monitoring duration for heart rate (hours); p:24-h period; $\bar{x}h$ : Mean heart rate during the h-th hour of each monitored day; x̄: Average heart rate per hour across the entire monitoring period; $\bar{x}i$ : Mean heart rate in the i-th hour; M10: Cumulative heart rate over the most active 10-h period; L5: Cumulative heart rate over the least active 5-h period.

### Criteria for classifying sleep–wake circadian rhythm

Phenotypes Circadian rhythm phenotypes were determined based on various sleep-phase parameters obtained from sleep monitoring data (Buysse et al., 1989). The classification criteria were as follows:

1. Increased sleep throughout the day and night: Total sleep duration (daytime + nighttime) > 10 h per day.
2. Reversed sleep–wake cycle: Daytime total sleep duration exceeds nighttime total sleep duration.
3. Increased daytime sleepiness: Daytime sleep duration >6 h per day.
4. Nocturnal sleep maintenance disturbance: Nighttime total sleep duration <5 h per day.
5. Difficulty initiating sleep: Nighttime sleep latency >30 min.
6. Reduced sleep efficiency: Nighttime sleep efficiency <75%.

### Statistical analysis

All statistical analyses were performed using SPSS version 26.0. Continuous variables with a normal distribution were expressed as mean ± standard deviation (x ± s). Group comparisons were conducted using independent-samples t-tests or one-way analysis of variance (ANOVA). Homogeneity of variance was tested via ANOVA; *post hoc* multiple comparisons were performed using the Bonferroni method. In cases of variance heterogeneity, Welch’s ANOVA was used along with the Tamhane method for multiple comparisons.

Non-normally distributed continuous data were expressed as median (interquartile range, Q1–Q3), and comparisons between groups were performed using the Kruskal–Wallis H test. Categorical variables were expressed as frequencies and percentages, and group comparisons were conducted using the chi-square (χ^2^) test.

Multiple linear regression was employed to adjust for potential confounding factors, while logistic regression was used to examine the relationships between circadian rhythm interdaily stability (IS), intradaily variability (IV), and the phenotypes of the sleep–wake rhythm. Given the small sample size, Firth regression was performed using the logistf package in R to correct the results.

## Results

### Comparison of baseline characteristics

There were no statistically significant differences between the hemorrhagic stroke group and the control group in terms of age, BMI, sex distribution, smoking history, or alcohol consumption (*p* > 0.05). However, significant differences were observed in the prevalence of hypertension, diabetes mellitus, hyperlipidemia, and hyperhomocysteinemia (*p* < 0.05) (Table 1).

**Table 1:** Comparison of baseline characteristics between the hemorrhagic stroke group and the control group.

Baseline characteristics	ICH group (*n* = 100)	Control group (*n* = 67)	*χ*^2^ (t)	*p*
Age (x̄ ± s)	63.23 ± 11.34	60.36 ± 9.67	−1.699	0.091
BMI (kg/m^2^)	24.22 ± 2.81	24.54 ± 2.44	0.779	0.437
Sex, *n* (%)			1.144	0.285
Male	72 (72.00%)	43 (64.20%)		
Female	28 (28.00%)	24 (35.80%)		
Smoking history, *n* (%)			0.009	0.926
No	53 (53.00%)	36 (53.70%)		
Yes	47 (47.00%)	31 (46.30%)		
Alcohol consumption history, *n* (%)			1.562	0.211
No	56 (56.00%)	44 (65.70%)		
Yes	44 (44.00%)	23 (34.30%)		
Hypertension, *n* (%)			54.757	0.000
No	8 (8.00%)	41 (61.20%)		
Yes	96 (91.4%)	26 (38.80%)		
Diabetes history, *n* (%)			5.531	0.019
No	71 (77.20%)	58 (86.60%)		
Yes	29 (29.00%)	9 (13.40%)		
Hyperlipidemia history, *n* (%)			4.440	0.035
No	83 (83.00%)	63 (94.00%)		
Yes	17 (17.00%)	4 (6.00%)		
Hyperhomocysteinemia history, *n* (%)			15.472	0.000
No	62 (62.00%)	60 (89.60%)		
Yes	38 (38.00%)	7 (10.40%)		

### Circadian rhythm analysis

Patients in the hemorrhagic stroke group exhibited significantly lower values for inter-daily stability (IS), intra-daily variability (IV), and relative amplitude (RA) compared to the control group (all p < 0.05), indicating disrupted circadian rhythmicity (Table 2).

**Table 2:** Comparison of circadian rest–activity rhythms between the hemorrhagic stroke group and the control group.

Circadian rhythm parameters	ICH group	Control group	Z/t	*p*
IS	0.31 (0.20, 0.53)	0.44 (0.31, 0.63)	−3.771	0.000
IV	0.59 (0.66, 0.94)	0.80 (0.66, 0.94)	−9.681	0.000
RA	0.34 ± 0.02322	0.40 ± 0.034	12.006	0.000

Adjustment for confounding factors: to control for the effects of potential confounding factors, we employed a multiple linear regression model to compare the differences in circadian rhythm parameters between the hemorrhagic stroke group and the control group, with adjustments for age, hypertension, diabetes, hyperlipidemia, and homocysteinemia. As shown in Table 3, after adjusting for the aforementioned variables, the hemorrhagic stroke group exhibited significantly lower IS (*p* < 0.001), IV (*p* = 0.002), and RA (*p* < 0.001) compared to the control group.

**Table 3:** Comparison of circadian rhythm parameters between the two groups after adjusting for confounding factors.

IS	*p*	OR (95%CI)	IV	*p*	OR (95%CI)	RA	*p*	OR (95%CI)
Group	0.001	−0.151 (−0.229 ~ −0.073)	Group	0.002	−0.175 (−0.284 ~ −0.065)	Group	0.001	−0.056 (−0.066 ~ −0.045)
Hypertension	0.227	0.051 (−0.032 ~ −0.135)	Hypertension	0.832	−0.013 (−0.13 ~ −0.105)	Hypertension	0.684	0.003 (−0.009 ~ 0.014)
Diabetes history	0.376	−0.034 (−0.108 ~ 0.041)	Diabetes history	0.877	−0.008 (−0.113 ~ −0.097)	Diabetes history	0.081	−0.009 (−0.02 ~ 0.001)
Hyperlipidemia history	0.873	0.008 (−0.086 ~ 0.101)	Hyperlipidemia history	0.494	−0.046 (−0.086 ~ −0.177)	Hyperlipidemia history	0.360	0.006 (−0.007 ~ 0.019)
Hyperhomocysteinemia history	0.19	0.049 (−0.024 ~ 0.122)	Hyperhomocysteinemia history	0.686	0.094 (−0.009 ~ −0.197)	Hyperhomocysteinemia history	0.106	−0.008 (−0.019 ~ 0.002)

^*^All variance inflation factor (VIF) values were below 2, indicating no substantial multicollinearity among the independent variables.

### Daytime sleep–wake monitoring results

**Figure 3:**
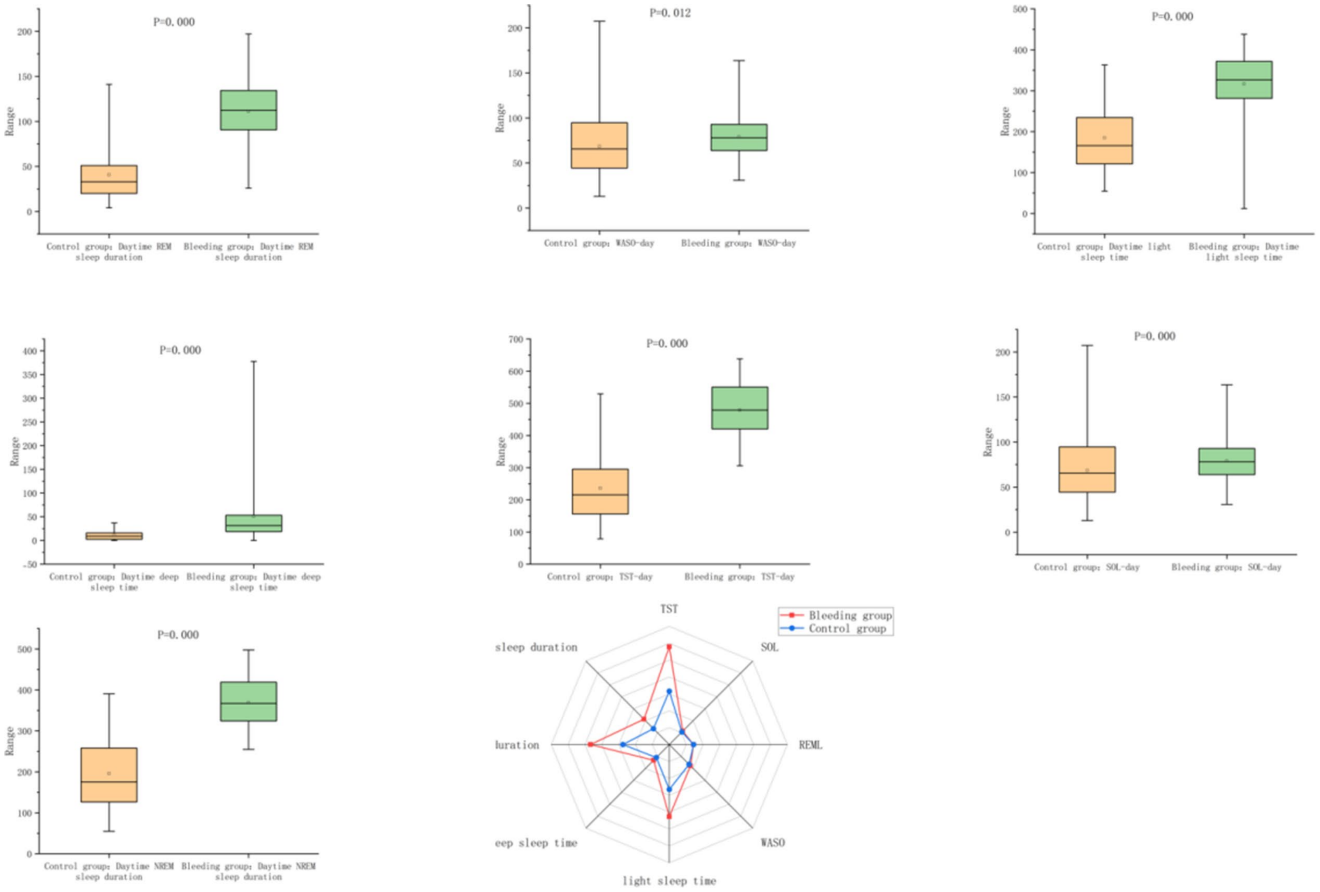
Comparison of daytime sleep–wake parameters between the hemorrhagic stroke group and the control group.

Compared to the control group, the hemorrhagic stroke group had significantly greater daytime total sleep time, durations of light sleep, deep sleep, NREM, and REM sleep, as well as higher sleep efficiency (all *p* < 0.05). Conversely, sleep latency, REM latency, and NREM percentage were significantly lower in the stroke group (*p* < 0.05). No significant difference was found between the two groups in daytime REM latency (*p* > 0.05) (Table 3; Figure 3).

### Nighttime sleep–wake monitoring results

**Figure 4:**
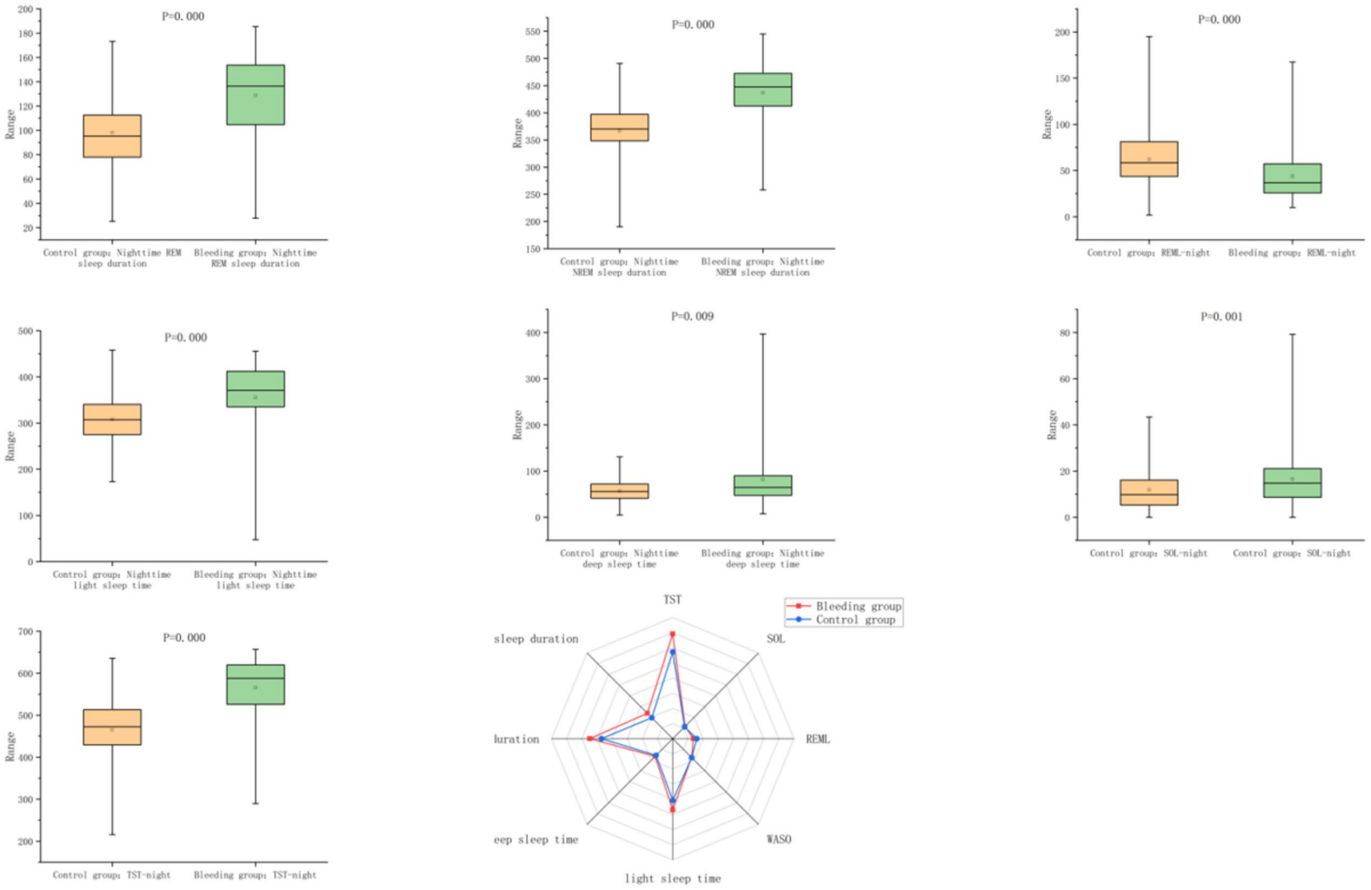
Comparison of nighttime sleep–wake parameters between the hemorrhagic stroke group and the control group.

During nighttime, patients in the hemorrhagic stroke group demonstrated significantly longer total sleep time and increased durations and percentages of light sleep, deep sleep, NREM, and REM sleep, along with higher sleep efficiency compared to controls (all *p* < 0.05). Meanwhile, sleep latency, REM latency, and NREM percentage were lower in the stroke group (*p* < 0.05). No significant group differences were found in wake after sleep onset, proportions of light and deep sleep, or overall sleep efficiency during the night (*p* > 0.05) (Table 4; Figure 4).

**Table 4:** Comparison of daytime sleep–wake parameters between the hemorrhagic stroke group and the control group.

Sleep metrics	ICH group (*n* = 100)	Control group (*n* = 67)	*Z*	*p*
TST-day (min)	478.93 (420.30,550.40)	215.80 (157.10,294.90)	−9.681	0.000
SOL-day (min)	12.47 (6.125, 17.47)	5.40 (2.40, 8.70)	−5.276	0.000
REML-day (min)	43.50 (29.00, 58.53)	44.20 (25.90, 62.60)	−0.029	0.977
WASO-day (min)	78.03 (63.84, 92.83)	65.60 (44.60, 94.30)	−2.522	0.012
Daytime light sleep time (min)	326.78 (281.25,371.60)	165.80 (123.20,233.80)	−8.070	0.000
Daytime deep sleep time (min)	31.53 (18.98, 53.20)	9.00 (2.60, 15.50)	−7.973	0.000
Daytime NREM sleep duration (min)	367.17 (324.33, 418.93)	175.20 (129.10, 258.10)	−9.400	0.000
Daytime REM sleep duration (min)	112.48 (90.60, 134.30)	33.00 (21.00, 50.00)	−9.172	0.000
Daytime Proportion of NREM sleep (%)	76.49% (74.31, 79.31%)	88.78% (81.40, 89.87%)	−6.827	0.000
Daytime Proportion of REM sleep (%)	23.37% (20.69, 25.69%)	15.41% (12.90, 18.64%)	−7.278	0.000
Daytime Proportion of light sleep (%)	69.75% (64.34, 72.77%)	80.43% (75.22, 84.40%)	−7.588	0.000
Daytime Proportion of deep sleep (%)	7.09% (4.27, 10.28%)	4.50% (2.58, 6.16%)	−4.663	0.000
Daytime sleep efficiency (%)	84.33% (81.81, 87.10%)	78.36% (73.58, 82.14%)	−6.413	0.000

### Comparison of sleep–wake circadian rhythm phenotypes

**Figure 5:**
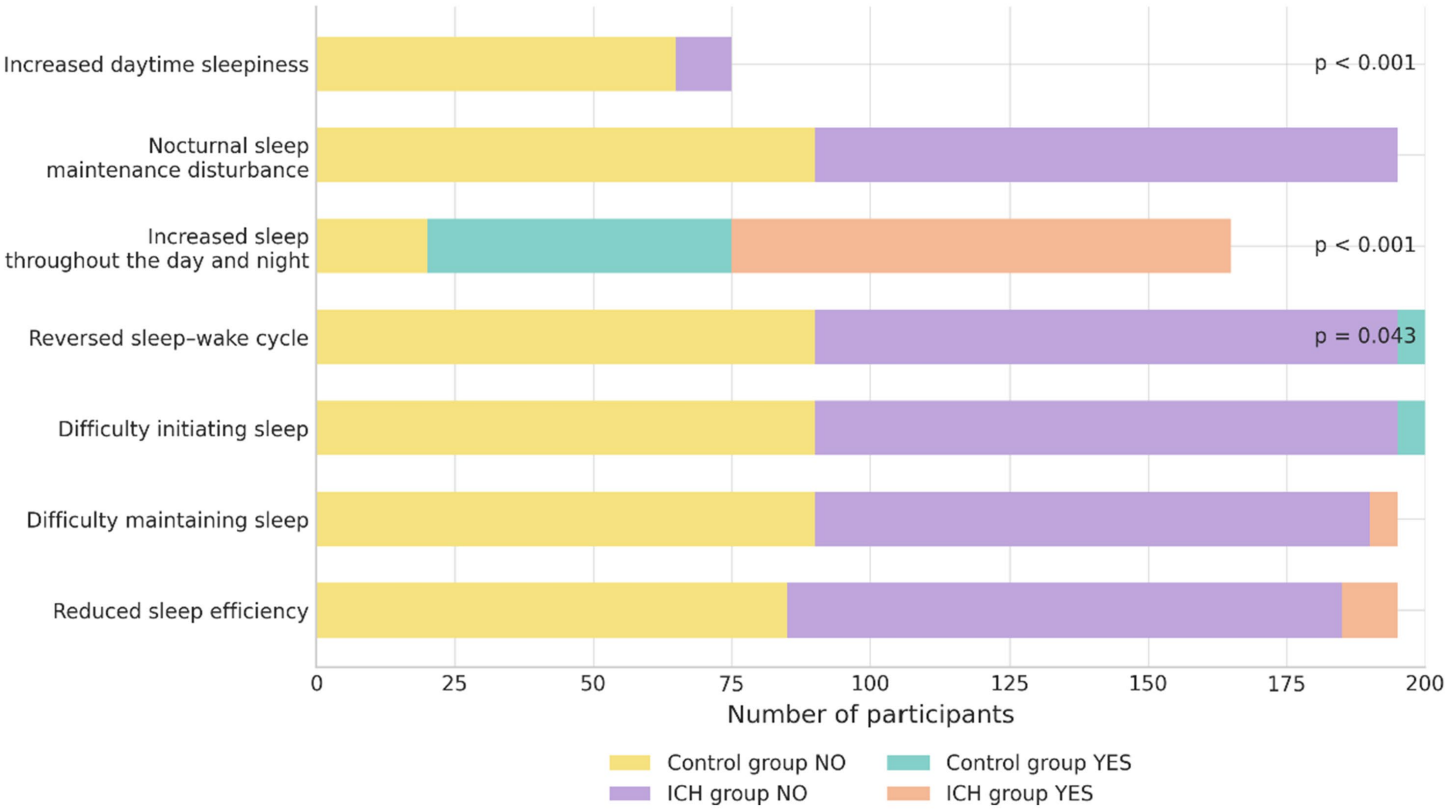
Prevalence of sleep disturbances in ICH and control groups.

The proportions of patients exhibiting excessive daytime sleepiness, global hypersomnia, and sleep–wake cycle inversion were significantly higher in the hemorrhagic stroke group than in controls (*p* < 0.05). However, the incidence of nighttime sleep maintenance disorder, difficulty initiating sleep, fragmented sleep, and low sleep efficiency did not differ significantly between the two groups (*p* > 0.05) (Table 5; Figure 5).

**Table 5:** Comparison of nighttime sleep–wake parameters between the hemorrhagic stroke group and the control group.

Sleep metrics	ICH group (*n* = 100)	Control group (*n* = 67)	*Z*	*p*
TST-night (min)	591.40 (527.17, 619.70)	472.40 (429.50, 508.90)	−7.36	0.000
SOL-night (min)	14.80 (8.63, 21.10)	10.40 (5.30, 16.10)	−3.193	0.001
REML-night(min)	36.70 (25.85, 57.17)	58.40 (43.60, 80.70)	−4.553	0.000
WASO-night(min)	75.00 (64.00, 106.40)	76.60 (64.00, 106.40)	−1.824	0.068
Nighttime light sleep time(min)	370.70 (335.03,412.10)	307.40 (275.80,338.20)	−6.009	0.000
Nighttime deep sleep time(min)	64.60 (47.48, 90.00)	55.80 (41.50, 71.90)	−2.617	0.009
Nighttime NREM sleep duration (min)	447.55 (412.50, 472.60)	370.40 (349.40, 397.00)	−7.436	0.000
Nighttime REM sleep duration (min)	136.33 (104.73, 153.80)	95.40 (79.10, 112.50)	−5.704	0.000
Daytime Proportion of NREM sleep (%)	77.08% (74.56, 80.28%)	79.46% (76.83, 81.59%)	−5.704	0.000
Nighttime Proportion of REM sleep (%)	22.92% (19.72, 25.44%)	20.79% (18.48, 23.10%)	−3.004	0.003
Nighttime Proportion of light sleep (%)	65.49% (61.32, 68.70%)	66.06% (62.20, 71.33%)	−1.215	0.225
Nighttime Proportion of deep sleep (%)	11.43% (8.76, 15.87%)	13.10% (9.16, 15.95%)	−0.552	0.581
Nighttime sleep efficiency (%)	86.19% (83.88, 88.50%)	85.72% (82.42, 87.74%)	−1.744	0.081

### Association between IS, IV, and sleep–wake circadian rhythm phenotypes in acute ICH

To explore the relationship between circadian rhythm parameters and specific sleep–wake phenotypes during the acute phase of hemorrhagic stroke, logistic regression analysis was performed using excessive daytime sleep and sleep–wake cycle inversion as dependent variables, and IS and IV as independent variables. Age was included as a covariate in the model to control for this confounding factor. The results indicated that IS was significantly associated with the presence of sleep–wake cycle inversion (Table 6).

**Table 6:** Comparison of circadian sleep–wake pattern types between the hemorrhagic stroke group and the control group.

Sleep pattern	ICH group (*n* = 100)	Control group (*n* = 67)	*χ* ^2^	*p*
Increased daytime sleepiness			91.198	0.000
Yes	89 (89.0%)	10 (14.9%)		
No	11 (11.0%)	57 (85.1%)		
Reduced sleep efficiency				0.565
Yes	1 (1.0%)	2 (3.0%)		
No	99 (99.0%)	65 (97.0%)		
Increased sleep throughout the day and night			21.041	0.000
Yes	100 (100.0%)	54 (80.6%)		
No	0 (0.0%)	13 (19.4%)		
Reversed sleep–wake cycle			4.105	0.043
Yes	11 (11.0%)	1 (1.5%)		
No	89 (89.0%)	66 (98.5%)		
Difficulty initiating sleep			0.097	0.755
Yes	9 (9.0%)	7 (89.6%)		
No	91 (91.0%)	60 (10.4%)		
Difficulty maintaining sleep				0.565
Yes	1 (1.0%)	2 (3.0%)		
No	99 (99%)	65 (97.0%)		
Reduced sleep efficiency				0.517
Yes	2 (2.0%)	0 (0.0%)		
No	98 (98.0%)	67 (100.0%)		

## Discussion

Our study found that patients in the acute phase of hemorrhagic stroke exhibited a state of profound hypersomnia, with a total sleep duration of 17.81 h per day. This finding aligns with alterations in circadian rhythms observed in this population. Compared to the control group, the stroke group demonstrated significantly lower inter-daily stability (IS), intra-daily variability (IV), and relative amplitude (RA), despite being in the same ward environment. This suggests that environmental or nursing-related physical factors alone cannot fully explain the observed disruption in the sleep–wake circadian rhythm (Table 7).

**Table 7:** Associations between inter-daily stability (IS), intra-daily variability (IV), and circadian sleep–wake pattern types during the acute phase of hemorrhagic stroke.

Increased daytime sleepiness (89^*^)	*p*	OR (95%CI)	Reversed sleep–wake cycle (11^*^)	*p*	OR (95%CI)
Age	0.893	−0.004 (−0.062 ~ 0.047)		0.546	0.019 (−0.478 ~ 0.087)
IS	0.621	−0.745 (−0.848 ~ 6.609)		0.007	−5.831 (−12.577 ~ −1.350)
IV	0.854	−0.193 (−2.131 ~ 0.047)		0.128	1.597 (−0.478 ~ 3.740)

^*^Values represent the number of patients; results were validated by Firth’s logistic regression.

The regulation of sleep–wake behavior is governed by both circadian and homeostatic mechanisms. Recent research has revealed that the neural circuitry involved in sleep–wake regulation includes multiple interconnected nuclei responsible for promoting either sleep or wakefulness, forming a complex and integrated regulatory network. This network is essential for maintaining the highly conserved and vital behavior of alternating sleep and wake states.

Key brain regions involved in sleep–wake regulation include the brainstem, hypothalamus, dorsal thalamus, and basal forebrain. These regions contain neurons that release various neurotransmitters, such as noradrenaline (NA), gamma-aminobutyric acid (GABA), acetylcholine, and histamine. These functionally distinct neuronal populations are primarily divided into two systems: the wake-promoting and sleep-promoting systems. These systems interact dynamically to regulate transitions between sleep and wake states (Scammell et al., 2017). The wake-promoting system comprises NA-releasing neurons in the locus coeruleus, serotonin (5-HT) neurons in the dorsal raphe nucleus (DRN), and neurons in the paraventricular nucleus of the dorsal thalamus (Ren et al., 2018). The sleep-promoting system includes GABAergic and galaninergic neurons in the ventrolateral preoptic area (VLPO) of the anterior hypothalamus (Saper and Fuller, 2017), as well as GABAergic neurons in the dorsal and ventral striatum and substantia nigra pars reticulata (Yuan et al., 2017; Liu et al., 2020; Oishi et al., 2017).

**Figure 6:**
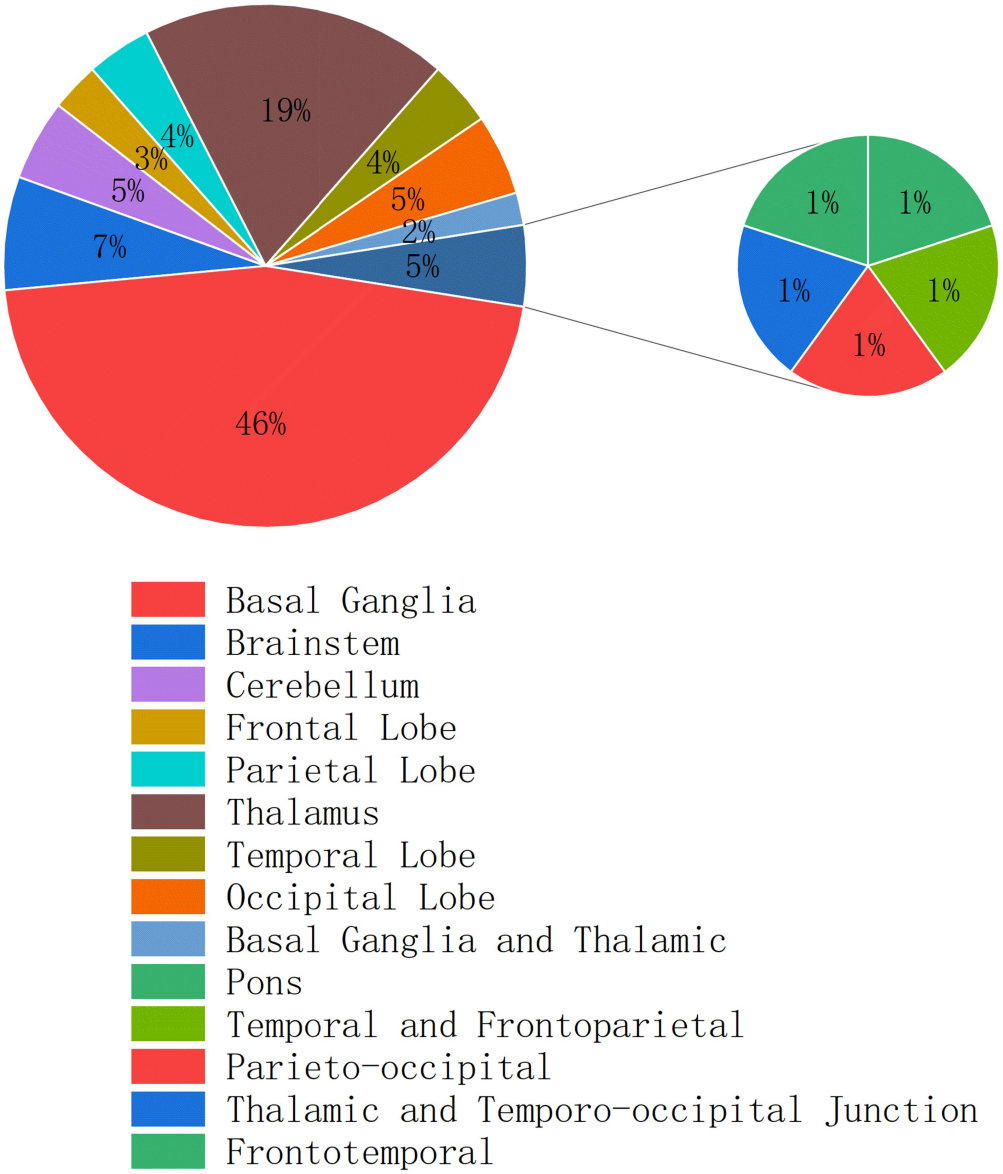
Distribution of hemorrhagic lesion locations in 100 patients with intracerebral hemorrhage.

In our cohort, the predominant hemorrhage sites were the basal ganglia and thalamus—regions intimately involved in sleep–wake regulation (Figure 6). Dopamine D1 receptor-expressing neurons in the dorsal striatum initiate and maintain wakefulness by integrating upstream inputs from the cortex, thalamus, and substantia nigra, and inhibiting sleep-promoting neurons in the globus pallidus internus and substantia nigra pars reticulata via GABA release (Dong et al., 2022). The dorsal thalamus also plays a pivotal role in modulating cortical oscillations during wakefulness and sleep. Optogenetic activation of the central medial nucleus of the dorsal thalamus has been shown to induce rapid transitions from non-REM (NREM) sleep to wakefulness (Gent et al., 2018). Moreover, calretinin-positive neurons in the mediodorsal thalamic nucleus regulate arousal levels by integrating subcortical inputs and synchronizing signals to the cortex (Mátyás et al., 2018).

We observed that the sleep architecture of hemorrhagic stroke patients reflected a hypersomnolent state, with prolonged durations across all sleep stages and shortened latencies to sleep onset, consistent in both daytime and nighttime recordings. These findings are supported by animal studies; one such mouse model (Giordano et al., 2020) reported post-hemorrhagic increases in sleep due to microglial activation and elevated pro-inflammatory cytokines, which can modulate sleep quantity and quality during recovery (Rowe et al., 2019).

When the sleep–wake regulatory network is disrupted, significant diurnal and nocturnal alterations in sleep–wake behavior are evident. In our study, patients exhibited increased durations and proportions of light sleep, deep sleep, NREM and REM stages, as well as elevated sleep efficiency. Conversely, sleep latency, REM latency, and the proportion of NREM sleep were reduced during both day and night (Figures 3, 4). These results are consistent with a recent study (Maas et al., 2020) using actigraphy, which showed that patients with intracerebral hemorrhage rapidly entered a state of behavioral quiescence, suggesting an adaptive brain response to severe acute stress that favors immobility rather than wakefulness—potentially beneficial for recovery. This hypothesis is supported by animal models exploring sleep under stress, which indicate that sleep plays a conserved role in mitigating cellular homeostatic disturbances (Hill et al., 2014). Moreover, a retrospective cohort study (Alabdali et al., 2025)reported that such hypersomnolence during the acute phase could persist into the rehabilitation period after discharge in a subset of patients, thereby adversely affecting quality of life and long-term outcomes.

This hypersomnolent state may also involve GABAergic neurons in the dorsal raphe nucleus (DRN), a key component of the sleep–wake regulatory network. The DRN contains serotonergic (5-HT), dopaminergic (DA), GABAergic, and glutamatergic neurons (Monti, 2010; Liu et al., 2020). GABAergic neurons are the second most abundant population and have been shown to play diverse roles in sleep–wake modulation (Sulaman et al., 2023). Specifically, glutamic acid decarboxylase 2 (GAD2)-positive neurons in the DRN (DRN^GAD2) are activated under acute stress in mouse models and suppress arousal by inhibiting wake-promoting neurons in the paraventricular thalamus (PVT) (Ren et al., 2024).

The circadian rhythm system regulates a wide array of physiological functions, including arousal, sympathetic tone, cardiovascular activity, coagulation, immune function, glucose regulation, and metabolism (Zaki et al., 2020; Nishimon et al., 2021; Coiffard et al., 2021). During critical illness, encephalopathy is a major risk factor for rapid central circadian disruption (Maas et al., 2020). By analyzing rest-activity rhythms (RARs), we found that IS, IV, and RA were significantly reduced in patients with hemorrhagic stroke, indicating a loss of rhythmicity in the activity-rest cycle and a blunting of circadian fluctuations. These results are consistent with the observed hypersomnolent state in these patients.

By examining rest–activity rhythms (RARs) under the same environmental conditions, we observed that patients with intracerebral hemorrhage (ICH) exhibited lower IS, IV, and RA values compared with controls. These findings indicate weakened synchronization with external environmental cues, flattened within-day activity–rest fluctuations, and an overall reduction in circadian differentiation and rhythm strength. This phenomenon suggests that peripheral rhythms have only limited autonomous maintenance capacity under acute stress, with their expression dependent on intact central–peripheral coupling.

ICH may disrupt the synchronization between the suprachiasmatic nucleus (SCN) and peripheral clocks through mechanisms such as increased intracranial pressure, systemic inflammatory responses, neuroendocrine activation (e.g., glucocorticoid and catecholamine release), and autonomic imbalance, thereby leading to circadian destabilization at behavioral and physiological output levels. In other words, although peripheral molecular clocks may continue to oscillate at low amplitude, the lack of effective central driving and integration with external zeitgebers prevents sustained behavioral rhythms.

Furthermore, prior studies in patients with anterior circulation ischemic stroke (ACIS) and posterior circulation ischemic stroke (PCIS) (Yuan et al., 2025) demonstrated that ACIS was associated with poorer daytime stability, greater circadian variability, reduced light sensitivity, and sleep–wake instability, whereas PCIS patients retained better daytime coordination and circadian recognition. These differences highlight distinct patterns of impairment in the central circadian pacemaker and its downstream neurovascular circuits. ACIS more frequently affects the anterior circulation and SCN, resulting in phase shifts and sleep–wake imbalance, while PCIS relatively preserves central driving and maintains entrainment to external cues. In contrast, ICH exacerbates SCN and downstream circuit dysfunction through acute intracranial hypertension, hematoma compression, widespread autonomic imbalance, and systemic inflammation, leading to consistent reductions in IS, IV, and RA. Collectively, this suggests that acute-phase ICH exerts a circadian impact at least comparable to, if not more severe than, acute ischemic stroke, driven by both focal neural damage and systemic stress–metabolic perturbations.

Moreover, the stroke group demonstrated a higher prevalence of increased daytime sleep, overall prolonged sleep, and reversed sleep–wake cycles. Circadian misalignment is closely linked to the development of sleep disorders (Yamanaka, 2020). To investigate whether acute disruptions in RARs are related to the emergence of sleep–wake disturbances, we conducted logistic regression analyses using IS and IV as independent variables and increased daytime sleep and reversed sleep–wake cycle as dependent variables. The results revealed that a lower IS was significantly associated with reversed sleep–wake patterns. This finding indicates that IS may serve as a non-invasive and observable indicator of clinical risk for sleep disturbances.

## Conclusion

Our study demonstrated that patients with acute ICH exhibited excessive sleepiness, disrupted sleep architecture, and attenuated circadian rhythms, characterized by significant reductions in IS, IV, and RA, as well as inverted sleep cycles. These results indicate that peripheral rhythms lack the capacity for independent maintenance under acute stress and rely on intact central–peripheral coupling. Multiple factors, including hematoma compression, intracranial pressure fluctuations, inflammatory responses, and autonomic imbalance, may jointly impair the circadian regulatory network. Clinically, such disturbances in sleep and circadian rhythms not only hinder neurological recovery in the acute phase but may also adversely affect long-term functional outcomes and quality of life. Future studies integrating circadian biomarkers (e.g., melatonin, orexin), genetic and molecular markers may further elucidate differential effects of ICH on central and peripheral rhythms, while personalized circadian-based interventions—such as light therapy, chronotherapy, and pharmacological modulation—could potentially improve sleep quality and neurological prognosis.

### Limitations

This study has several limitations. First, as a retrospective study, its conclusions may be influenced by selection bias and unmeasured confounding factors. Second, rest-activity rhythm monitoring was performed using a mattress-based heart rate system rather than polysomnography, which may reduce measurement accuracy. Additionally, the monitoring duration was less than 7 days. Although focusing on the acute phase is reasonable, the short monitoring period may be insufficient to clearly distinguish between circadian rhythm dysfunction caused by stroke itself and transient rhythm fluctuations due to acute physiological stress. Extending the monitoring duration would help improve the robustness of the conclusions. Furthermore, medication history was not systematically recorded, making it impossible to exclude the potential influence of drugs on circadian rhythms. The limited sample size and the exclusion of patients over 75 years of age also restrict statistical power and generalizability to older populations. Moreover, the study did not utilize light sensors and lacked molecular mechanism data (e.g., clock genes, melatonin), which should be addressed in future research. Despite these limitations, this study provides valuable clinical evidence regarding circadian rhythm alterations in patients with acute intracerebral hemorrhage.

## Data Availability

The raw data supporting the conclusions of this article will be made available by the authors, without undue reservation.
